# The Complex Relationship Between Tuberculosis and Hyperglycemia

**DOI:** 10.3390/diagnostics14222539

**Published:** 2024-11-13

**Authors:** Michelle Byers, Elizabeth Guy

**Affiliations:** Section of Pulmonary Critical Care and Sleep Medicine, Baylor College of Medicine, Houston, TX 77030, USA

**Keywords:** tuberculosis, hyperglycemia, diabetes, pre-diabetes, intermediate hyperglycemia

## Abstract

Hyperglycemia and tuberculosis are dual global pandemics. Each has a propulsive and amplifying effect on the other, and, because of this, we must consider hyperglycemia and tuberculosis together. Hyperglycemia is immunosuppressive and increases the risk of tuberculosis by threefold. It also leads to a more advanced presentation of pulmonary tuberculosis, thus increasing the likelihood of being smear positive and having cavitating lesions, and it impacts the duration and outcomes of treatment, with an increased one year mortality seen in patients with tuberculosis and diabetes. Additionally, any degree of hyperglycemia can have an impact on susceptibility to tuberculosis, and this effect is not limited to poorly controlled diabetes. Conversely, tuberculosis itself is associated with hyperglycemia and worsens hyperglycemia in those with diabetes mellitus. The impact of this relationship varies based on the base rates of each disease in different regions of the world. In order to successfully achieve the World Health Organization’s goals of tuberculosis eradication and adequate glycemic control, we must improve our understanding, co-management, and screening of hyperglycemia and tuberculosis. This review aims to explore the current research investigating the relationship between tuberculosis and diabetes, including the changes in disease susceptibility, presentation, geographic distribution, and effects on treatment.

## 1. Introduction

Tuberculosis (TB) and hyperglycemia have had vast effects on human health for centuries. Although these two diseases are not frequently thought of together, their paths are in fact not parallel but better characterized as circuitous intersections that often converge. Now, in our modern times, this convergence is even more apparent and important to recognize.

An estimated one quarter of the human population is infected with TB, with 1.3 million people dying from TB in 2022. TB ranked second among worldwide causes of death from infection, below COVID-19 but above HIV [1]. Globally, approximately 10.5% of adults have diabetes mellitus [2]. By examining the relationship between these two diseases through a meta-analysis of 13 studies, Jeon et al. [3] showed that patients with diabetes have a threefold increased risk of tuberculosis disease (TB) when compared to patients without diabetes. This highlights a large population of people who are at increased risk but who are not often recognized by the general medical community. This recognition offers an opportunity for targeted interventions to improve disease rates and outcomes for patients with TB and diabetes (TB-DM).

The incidence of TB is slowly trending downwards. However, the incidence of diabetes is increasing year to year. For example, in the Unites States, the prevalence of diabetes is projected to increase between 2015 and 2030 by 54%, resulting in 54.9 million Americans with diabetes [4]. Sung-Chin Pan et al. [5] performed a modeling study of the effects of the rise of diabetes on the incidence of TB. They found that at the current rate of decline of tuberculosis (about 2% per year) and with the projected increase in the prevalence of diabetes, TB incidence should decrease by 8.8%. However, if the prevalence of diabetes could be lowered by 6.6–13.8%, the reduction in TB incidence could be as much as 11.5–25.2%. If this scenario could be achieved, it would prevent 6 million cases of TB and 1.1 million deaths secondary to TB. This illustrates the dramatic association between these diseases as well as the importance of dual control of diabetes and cure of tuberculosis. However, the relationship between hyperglycemia and tuberculosis is not simple or linear. There is a complex interplay between the two disease states rather than one having a causative effect on the other. In this review, hyperglycemia refers to any degree of hyperglycemia, including intermediate hyperglycemia, pre-diabetes, and diabetes mellitus. The studies cited used different tests to classify hyperglycemia, and the results varied based on the test. Type 1 DM occurs in 5% of patients with DM, and clinical studies do not make a distinction between type 1 and type 2 DM. We will explore the current research on the relationship between tuberculosis and diabetes to better understand and define this association.

## 2. Effect of Hyperglycemia on Contracting Tuberculosis

### 2.1. Why Diabetes Is Immunosuppressive

Jeon et al. [3] established that patients with diabetes have a 3-fold increased risk of TB. This effect was re-established in an updated meta-analysis in 2021 showing an odds ratio of 2.3 that was sustained across cohort, case control, and cross-sectional studies [6]. This prompts the following question: why do hyperglycemia and diabetes increase a person’s risk for TB? Diabetes affects several pivotal moments within the immune system’s processing and response to *Mycobacterium tuberculosis* (*M.tb*). The causes of this damaged immune system are hyperglycemia and insulin deficiency. The known mechanisms of immunosuppression related to hyperglycemia are reduced cytokine production, defective recruitment of leukocytes, impaired pathogen recognition, dysfunction of neutrophils, macrophages, and natural killer cells, and inhibition of complement activation, causing downstream inhibition of antibodies. This extensive list involves many aspects of both the innate and the adaptive immune response to *M.tb*, and is briefly described below [7]. Apart from the innate and adaptive immune defects caused by diabetes, other pathways of immune suppression are beyond the scope of this review.

### 2.2. Why Diabetes Increases Susceptibility to Tuberculosis

The natural course of TB infection is as follows. Bacilli are inhaled into alveolae, where they infect local macrophages, which, if they fail to kill the bacteria, results in bacterial multiplication and apoptosis of the macrophages. Released bacilli are taken up by other immune cells, such as dendritic cells. The dendritic cells carry the bacilli to the thoracic lymph chain and prime the adaptive lymphocyte response, resulting in T-cell-mediated immunity directed back into the alveoli. Memory T helper 1 cells release IFNγ to further activate the immune system, resulting in granuloma formation to restrict the bacteria. After this, the body takes one of three pathways: it completely clears the bacteria, the granuloma forms, but it does not completely kill the bacteria, resulting in latent TB infection (LTBI), or, if there is a failure to control the pathogen, the patient develops active TB [8,9].

Diabetes and hyperglycemia increase susceptibility to TB due to effects on both the innate and the adaptive immune responses. The innate immune system includes alveolar macrophages, neutrophils, dendritic cells, and innate lymphoid cells, among others. Vrieling et al. [10] found that oxidized LDL, which is highly elevated in diabetes, caused significantly elevated levels of *M. tb* within macrophages. This was related to lysosome dysfunction, resulting in macrophages being unable to kill the bacteria and instead allowing it to proliferate. Hyperglycemia also decreases antigen processing and presentation in macrophages [11]. Macrophages in TB-DM patients not only produced lower levels of pro-inflammatory cytokines but also produced increased levels of anti-inflammatory cytokines, such as IL-10 [12]. This slow response defers activation of the adaptive or cell-mediated immune response, which in turn allows for early TB growth, thus disrupting the very first step in immune TB processing [13]. In vitro studies have shown that in TB-DM, monocytes have decreased recognition and phagocytosis of *M.tb*, which prevents monocytes from activating downstream immunogenic pathways. Additionally, this highlights the connection between diabetes, metabolic syndrome, and tuberculosis susceptibility. Two other major cell responses in the innate immune system involve neutrophils and dendritic cells. Interestingly, in TB-DM patients, the absolute number of neutrophils is increased, but they have reduced phagocytic capabilities [14]. This paradox highlights the overall immune response in TB-DM patients: an increased inflammatory response with a decreased functional response [15]. The dendritic cells are the bridge between the innate and adaptive immune systems. In TB-DM patients, there is a decrease in the quantity of dendritic cells when compared to non-TB-DM patients. Importantly, there is also a negative correlation between hyperglycemia and dendritic cell subset frequency, indicating that the dysfunction likely occurs not only in T2DM but also in patients with intermediate hyperglycemia. This effect was also seen with monocytes. Both cell lines showed normalization after completing TB treatment [16].

The adaptive immune response in the setting of hyperglycemia is more complex, as different aspects are downregulated and upregulated, resulting in an unbalanced response that leads to *M. tb* growth and potential dissemination. The adaptive immune response is categorized into Th1-, Th2-, or Th-17-type responses. The ideal immune response to TB is Th1 characterized by inflammation, with IFNγ, IL-2, and TNFα targeting intracellular organisms. Wang X et al. demonstrated that while there is no difference in Th1 stimulation in TB patients with or without DM, there was a significant increase in Th2 stimulation in TB-DM compared to those without DM. However, they also found that the overall activity of CD8+ cells (Th1 response) was decreased in TB-DM patients [17].

Studies have reported conflicting results regarding which cytokines are increased or decreased in TB-DM patients. Eckold et al. [18] used RNA sequencing to analyze alterations in gene expression in patients with euglycemia, intermediate hyperglycemia (defined based on a measurement of glycated hemoglobin or HbA1c of 5.7–6.4), and patients with diabetes, all in the setting of tuberculosis. Their findings demonstrated hyperexpression of the inflammatory response and suppression of the IFNγ pathways in both the diabetes and the intermittent hyperglycemia groups. However, in a study performed in the US National Health and Nutrition Examination Survey, IFNγ was increased in both DM and pre-DM patients. These patients also had a higher incidence of TB compared to the non-DM population [19]. Although these studies show inconsistent patterns of cytokine levels in TB-DM patients, they reinforce the dysregulated hyperinflammatory yet ineffective immune response present in patients with DM as well as those with pre-DM or intermediate hyperglycemia. The study populations also differ, as Eckold et al. [18] studied populations in South Africa, Romania, Indonesia, and Peru, whereas Magee et al. [19] studied US patients.

There is burgeoning research on mechanisms and pathways of gene and protein expression that help to explain how hyperglycemia causes higher rates of TB infection and active disease that is beyond the scope of this review [9,20].

## 3. Bi-Directional Relationship

Tuberculosis and hyperglycemia can be interdependent, which prompts the following question: which disease is a cause, and which is an effect? Studies have shown that the cytokine release caused by tuberculosis activates the hypothalamic–pituitary axis. The resulting increased release of cortisol, catecholamines, epinephrine, norepinephrine, thyroid releasing hormone, and growth hormone may lead to stress-induced hyperglycemia [20]. Cortisol levels decreased by week 4 of treatment in patients who achieved treatment success and were maintained at low levels; however, for those patients who failed treatment, the cortisol levels remained elevated [21]. Adult mice infected with TB fed a high-fat diet had significantly increased levels of insulin compared to uninfected mice, with a high-fat diet raising concerns about TB inducing insulin resistance [22].

Epidemiologic studies show that tuberculosis can lead to hyperglycemia, as seen in multiple studies documenting the presence of intermediate hyperglycemia at the time of TB diagnosis [23,24,25]. A high proportion will experience reversal of hyperglycemia after treatment of TB [21,24]. A case control study in Tanzania demonstrated that in a population with a low prevalence of diabetes, hyperglycemia at the time of TB diagnosis is more likely to be related to infection-stress-induced hyperglycemia than true diabetes. At follow-up, between 59 and 100% of patients who had hyperglycemia were normoglycemic based on the type of test used to define hyperglycemia/DM [24]. In contrast, in Pakistan, where rates of diabetes are high, Aftab et al. [21] reported that in a cohort of patients with TB, screening for DM at diagnosis showed that 42% had a fasting plasma glucose (FPG) > 7 mmol. At the 3- and 6-month follow-ups, 46% and 62%, respectively, were normoglycemic. Menon [23] also noted that three screening tests used to diagnose DM had a concordance of 22% at enrollment and 53% at follow-up. Kumpatla and colleagues [26] studied fasting plasma glucose and HbA1C to define DM in a cohort of 779 TB patients and found that HbA1C performed better than FPG [26]. Thus, the diagnostic test used to screen for DM may explain the heterogeneity of the results.

The trajectory of hyperglycemia after treatment of TB is heterogeneous, with studies showing an overall improvement in hyperglycemia, including those with preexisting diabetes. However, somewhere between 10 and 50% continue to have hyperglycemia after TB treatment [23,27,28]. This raises the possibility that a prior episode of TB may be a risk factor for the development of DM, much like gestational diabetes. In a systematic review and meta-analysis of outcome studies in high-TB-burden countries, including India, Indonesia, China, the Philippines, Pakistan, Nigeria, and South Africa, the pooled unresolved hyperglycemia at 3–6 months was 11% [23]. In addition, there are epidemiologic studies that suggest this possibility. Magee [29] studied the US Veterans Health Administration database of more than half a million patients who were tested for TB infection (Tuberculin Skin Test or Interferon Gamma Release Assay IGRA) between 2000 and 2015. They reported a higher incidence rate of DM, with a hazard ratio of 1.4 (95% confidence interval 1.3–1.4) among those who had latent TB infection compared to those without. In a 12-year study from Taiwan, the age-adjusted incidence rate of DM was 3.85 (95% CI 3.7–4) among those who had TB, with a median time of 7 years after diagnosis [30]. Other cohort studies from Denmark, United Kingdom, and Guizhou, China showed a positive correlation between pulmonary tuberculosis and a new diagnosis of diabetes [31,32,33].

## 4. Effect of Geography on TB-DM

While diabetes and tuberculosis are a global syndemic, the nature of their interaction is highly variable depending on the geographic location. A recent systematic review and meta-analysis looked at 200 different studies to further elucidate the range of impact these diseases have depending on the region [34]. The global prevalence of TB-DM was 15.3%, with significant heterogeneity. Their findings demonstrate that the highest co-existence was shown in the International Diabetes Federation (IDF)-defined regions of North America and the Caribbean, Southeast Asia, the Middle East, and North Africa compared to lower co-prevalence in South and Central America, Africa, and Europe. Notably, the prevalence of diabetes in patients with tuberculosis was higher in areas with an overall low prevalence of tuberculosis compared to those with a high prevalence of tuberculosis. The study considered the relative imbalance between the high burden of HIV in low-income countries and developed countries, which is the inverse of diabetes’ prevalence. This suggests that in developed nations, diabetes could be a more important risk factor driving tuberculosis, even though, globally, HIV is number one [34]. Reinforcing this is a study at the Texas/Mexico border. In south Texas, the fraction of tuberculosis attributable to diabetes was 63%, and it was 68% in northeastern Mexico. In contrast, the fraction of tuberculosis attributable to HIV in these populations was <5.0%. Notably, when looking at individuals in these areas who were HIV positive, HIV infection accounted for 94% of tuberculosis [35]. This reinforces that on a population level, diabetes, when highly prevalent, is a major driver of tuberculosis.

As demonstrated by the different locations and findings of these studies, the strength and directionality of the relationship between TB and DM is likely dependent not only on the region but also on the burden of each disease in the area. In a systematic review, Workneh [36] reported on the prevalence of DM among TB patients and vice versa. The prevalence indeed varied by geographic region, as shown in the Table 1. A noteworthy conclusion is that the prevalence of TB in DM patients was low, ranging from 0.38 to 14% globally. A prevalence of 0.38% translates into 380 cases/100,000 people with diabetes. This is a staggering number, as the World Health Organization (WHO) defines a high TB burden setting when TB incidence is >100/100,000 population [37]. This reinforces the need to approach TB-DM by region.

## 5. Degree of Hyperglycemia Required

An increased prevalence of TB and an impaired immune response to TB can be seen with intermediate hyperglycemia or pre-diabetes, and this prevalence is similar to that reported for diabetes mellitus [38,39]. However, is the risk of TB equal regardless of the degree of hyperglycemia, or is it a linear relationship? Epidemiologic studies show that patients with intermediate hyperglycemia or pre-diabetes have a higher risk of tuberculosis, and they are more likely to be sputum smear positive than normoglycemic TB patients [40]. In a mouse model, those with pre-diabetes had increased lung pathology and lower concentrations of Th1 inflammation (IFNγ, TNFα). Interestingly, higher body fat with preserved glucose tolerance was protective [41]. A prospective cohort study in Taiwan showed a positive relationship between the number of diabetes-related complications and the risk of TB, which suggests that while the risk begins with intermediate hyperglycemia, it likely increases with more severe hyperglycemia [42]. Another study of individuals in Hong Kong showed a 2.5 hazard ratio of active TB when comparing diabetics with HbA1c > 7.0 compared with diabetics with HbA1c < 7.0, again demonstrating a potential “dose effect” of hyperglycemia on TB risk [43]. Patients with higher levels of fasting plasma glucose levels and HbA1c are more likely to be sputum positive, have pulmonary lesions, and more cavitary lesions when compared to diabetic patients with better glycemic control [44]. Additionally, the degree of hyperglycemia had a weakly positive association with a high CT score for TB-DM patients [45].

## 6. Effect of Hyperglycemia on Presentation of Tuberculosis

It has been well-established that TB-DM patients, when compared to non-DM patients, are more likely to be sputum smear positive [46,47,48,49,50], with pulmonary vs. extrapulmonary disease [43] and cavitating lesions [51,52], and they have a higher bacillary load [53]. A meta-analysis also showed an increased risk of LTBI in patients with diabetes, with an adjusted odds ratio of 1.21 and a crude odds ratio of 1.64 [54].

Additionally, close contacts of persons with TB and hyperglycemia are more likely to become infected with TB. An interesting study in Brazil monitored close contacts of patients with TB using QuantiFERON TB positivity status at baseline and after 6 months. The subjects were stratified by their contact with a person with TB-DM vs. TB alone. Importantly, the study included persons with pre-DM as well as diabetes. Pre-DM alone was a risk factor for close contacts being QuantiFERON TB positive at baseline. TB-DM was a risk factor for conversion from QuantiFERON TB negative to positive after 6 months. Notably, “close contact” was defined as >4 h contact per week [55]. This has significant public health implications for attempts at controlling the spread of TB, especially in populations with a high incidence of DM.

## 7. Effect on Treatment Response

TB-DM patients take longer to convert from smear positive to smear negative [51]. Additionally, TB-DM patients are more likely to have multi-drug-resistant (MDR) tuberculosis. Among TB-DM patients with MDR TB, independent risk factors included age < 65 and HbA1c level. A level of HbA1c of 9.3 was associated with a higher prediction value of MDR TB [56].

TB-DM patients were also at a significantly higher risk of treatment failure, with a hazard ratio of 0.46 [57,58]. A prospective case control study in Taiwan found an increased risk of pulmonary tuberculosis recurrence in TB-DM patients with an odds ratio of 1.67 [59]. TB-DM patients had an 8.9-fold risk of treatment failure in a study from Armenia [58].

TB-DM patients are more likely to have unfavorable treatment outcomes, defined as treatment modification, treatment failure, recurrence, or death during TB treatment. In a systematic review of 33 studies, Baker [51] found that the unadjusted relative risk of death was 1.89, and it increased to 4.95 when adjusted for age and other confounding factors. The impact of diabetes on mortality was found to be greater in patients younger than 50, and it occurred during the 6 months following treatment in a cohort of 657 TB patients in South Korea [60]. Faurholt-Jepsen [48] also found a 5-fold higher mortality rate in TB-DM patients who were HIV negative during the first 100 days of treatment compared to a twofold mortality rate among those who were co-infected with TB-HIV.

Mave and colleagues [61], in a prospective study of treatment outcomes in India, found that while the likelihood of a poor overall outcome was no different between DM and non-DM patients, early mortality during treatment was higher in TB-DM patients. In a Brazilian multicenter prospective cohort study, those with DM and not pre-DM were more likely to experience treatment modification, failure, or death. The study also found that TB-DM is associated with a higher relative risk of death [62].

## 8. Factors to Consider During Treatment

As mentioned, Oswal et al. [22] demonstrated that TB infection can induce insulin resistance. Their data indicate that the etiology of this is likely related to dysregulation of adipocytes, lipolysis, and lipid metabolism due to adipocytes being a reservoir for TB bacilli [22]. Treatment for TB has been shown to improve hyperglycemia and glycemic control [63]. The Collaborative Framework for the Care and Control of Tuberculosis and Diabetes by the WHO and the Union (the International Union Against Tuberculosis and Lung Disease) in 2011 recommends high-quality management of diabetes in patients with tuberculosis [64]. A systematic review and meta-analysis showed reduced sputum positivity and cavitary lesions in patients with optimal glycemic control when compared to those with suboptimal glycemic control [64,65]. However, there is no guidance on the optimal glycemic control target for TB-DM patients, treatment for tuberculosis can improve insulin resistance, which raises the possibility of iatrogenic hypoglycemia if strict euglycemia is targeted compared to a more liberal goal. However, this has not been specifically tested.

During TB and DM treatment, a variety of drug interactions must be considered. Rifampin is a potent CYP3A4 inducer. This predominantly affects diabetes care for those on sulfonylureas by inducing rapid hepatic clearance of the drug, thus making it less efficacious. However, other studies have found that it has no effect on the oral hypoglycemic medication class including glucagon-like peptide-1 agonists (GLP-1), and a very small effect on another oral hypoglycemic medication class, Dipeptidyl peptidase 4 inhibitors (DPP4 inhibitors), so not all oral hypoglycemics are impacted [66].

Independent of drug interactions, it has also been noted that the Tmax (time for a drug to reach its maximum concentration following administration) for rifampin is prolonged in TB-DM patients. The maximum concentration of the drug is not significantly different between TB-DM and TB patients; however, the delay in reaching maximum concentration suggests the need for monitoring of rifampin levels early in the treatment of TB-DM [67].

Metformin may also confer some benefits in TB-DM patients. In vitro and mouse studies of metformin have shown that it may increase the macrophage autophagy of TB and decrease the pulmonary bacillary load. In a retrospective study of patients with TB-DM in Singapore, those on metformin had fewer pulmonary cavities, and mortality was 3% vs. 10% in the non-metformin group [68]. While the later data merely showed an association, the group’s former laboratory studies demonstrated a possible causal relationship between metformin and improved outcomes. In a prospective cohort study in west China, TB-DM patients on metformin were more likely to be smear negative after two months of anti-TB therapy compared with those with TB-DM not on metformin [69]. Statins have also shown some benefit in this population of patients. *M. tb* utilizes cholesterol to invade macrophages, and animal studies have shown some decreased *M. tb* burden in subjects on statin therapy. Additionally, some retrospective studies show a protective effect of statin therapy against active TB. However, no prospective studies have been conducted to help strengthen this assertion [70]. Another cross-sectional study showed significantly reduced levels of LTBI in patients using metformin and statin, with a prevalence of less than half compared to those with diabetes who were not on either medication [71]. Much more research must be performed before any real conclusions can be made, but this is a potentially promising drug effect in the co-management of these diseases.

## 9. Screening Programs

The WHO Global Tuberculosis Report in 2023 listed five risk factors for new cases of tuberculosis: undernourishment, HIV infection, alcohol use, smoking, and diabetes [1]. As far back as 2011, the WHO has recommended collaborative care for TB-DM patients, culminating in the publication of the Collaborative Framework for the Care and Control of Tuberculosis and Diabetes, outlining the three main pillars of their guidelines: (1) establish mechanisms of collaboration, (2) detect and manage tuberculosis in patients with diabetes, and (3) detect and manage diabetes in patients with tuberculosis. Since these recommendations were made, the 2021 Global Tuberculosis Report reviewed the tuberculosis policies of 30 nations with the highest tuberculosis burden [72]. Many countries made reference to the need for screening for TB in patients with diabetes, but only two (Tanzania and South Africa) actually created programs for joint screening and co-management of the diseases [72]. When creating a screening program, whether unilateral or bidirectional, feasibility and utility must be considered. In a recent study in Malawi, workers at both TB-DM integrated care facilities as well as workers at non-integrated facilities were polled on the perceived practicality of a collaborative care model for TB-DM patients. In total, 94% of those already working at integrated facilities thought it was feasible vs. 86% of those at non-integrated facilities. This small study demonstrated that while the prospect of this kind of care can be daunting, it is less so to those who have already begun to undertake it [73]. A similar style of study in Indonesia found that patients with diabetes were mostly happily accepting of tuberculosis screening, with patient buy-in being an important factor in a screening program. This openness to screening was not universal, though, as some patients demonstrated reluctance towards screening due to social stigma surrounding tuberculosis. Potential operational barriers noted were the burden of increased clinical decision making and the need for referrals to treatment centers with little knowledge of the patient’s history [74]. A cross-sectional study across Indonesia, Romania, Peru, and South Africa investigating screening for tuberculosis in diabetes patients (the screening method included a symptom questionnaire and a chest X-ray) found 14 new cases of diabetes out of 2063 patients screened. Interestingly, 13 out of the 14 cases were identified based on symptoms alone, and the chest X-ray was not useful in screening [75].

In a study examining a population-based screening program for tuberculosis in diabetes patients in Jiangsu, China, the number needed to screen (NNS) to diagnose tuberculosis was 560. The cost per screening was expensive at USD 13,930; however, in those with symptoms of TB, the cost decreased to USD 1037. They also performed a systematic review and a meta-analysis of population-based tuberculosis screening programs in patients with diabetes. NNS was unsurprisingly but notably different when examined in high vs. low tuberculosis burden areas. In high TB burden areas, it was 93 vs. 395 in low TB burden regions [76]. This again highlights the need to tailor not only treatment systems but screening programs to the patterns seen within a region, as a single global screening protocol would be impractical and ineffective.

When considering screening for diabetes in those with TB, there are even more complications to consider. There are some data that suggest that due to infection-induced hyperglycemia, as discussed earlier, it would be more beneficial to screen for diabetes later in the tuberculosis treatment course [49]. A cross-sectional study examining NNS to prevent an adverse event or death in patients with known pulmonary tuberculosis in Guizhou, China found that the NNS was 359. To put the NNS into context, the generally acceptable NNS for cancer screening ranges from 500 to 1000. All of the screening programs evaluated above for DM in TB patients and vice versa fall well below this range [77].

In 2023, in the United States, the US Preventative Services Task Force released a recommendation statement on screening for LTBI in the US in which they recommended screening “at risk” patients. Who they deem at risk was first based on local health department data and then on those with known tuberculosis risk factors, such as those born in nations with a high prevalence and those who spend time in high-risk congregate settings. They discuss the known risk of TB infection in persons with diabetes; however, they do not make a specific recommendation due to insufficient evidence [78].

## 10. Conclusions

Tuberculosis and hyperglycemia have a complex, interdependent relationship that ultimately results in a higher burden of disease and death for patients. Hyperglycemia with or without true diabetes causes immunosuppression that is uniquely effective at facilitating TB infection. Intermittent hyperglycemia or pre-diabetes has not been a focused area, but it is an important group to incorporate into our understanding of tuberculosis susceptibility. At this time, we have a better understanding than we had in prior decades about how and why these diseases portend poor outcomes for patients. We do not yet know (a) if there is a true causal relationship, (b) how to practically and effectively screen for these diseases in patients in a regionally directed manner, or (c) if treatment of these patients must be adjusted based on disease co-existence. To achieve the WHO goal to end the global TB epidemic by 2035, we need to focus our future research on interventions for this cohort of patients.

## Figures and Tables

**Table 1:** Prevalence of DM among TB patients and prevalence of TB among DM patients [36].

	Number of Studies	DM in TB Patients, %	Median (IQR)	Number of Studies	TB in DM Patients, %	Median (IQR)
Globally	78	1.6–45	16 (9–25.3)	19	0.38–14	4.1 (1.8–6.2%)
Asia	48	5.1–44	17 (11.4–25.8)	10	0.38–14	3.5 (0.9–10.5)
Africa	12	1.9–16.7	6.7 (4.1–10.4)	7	1.3–6.2	5.6 (3.5–5.8)
Oceania	5	12–45	23.2 (12.8–39)			
North America	8	11.4–39	23.6 (17.3–35.4)	1	4.9	
South America	3	6.1–14	11.1 (6.1–14)			
Europe	1	5.9		1	1.82	

## Abbreviations

TB: tuberculosis; TB-DM: patients with both tuberculosis and diabetes; M.tb: Mycobacterium tuberculosis; IFNγ: interferon gamma; LTBI: latent tuberculosis infection; LDL: low density lipoprotein; T2DM: type two diabetes; TNFα: tumor necrosis factor; HbgA1c: glycolated hemoglobin; pre-DM: pre-diabetes; WHO: World Health Organization; FPG: fasting plasma glucose; TST: tuberculin skin test; IGRA: inferno gamma release assay; IDF: International Diabetes Federation; CYP4A4: cytochrome P450 C4; NNS: number needed to screen.
